# Vole outbreaks may induce a tularemia disease pit that prevents Iberian hare population recovery in NW Spain

**DOI:** 10.1038/s41598-023-30651-7

**Published:** 2023-03-08

**Authors:** Carlos Rouco, Juan José Luque-Larena, Dolors Vidal, François Mougeot

**Affiliations:** 1 Área de Ecología, Departmento de Biología Vegetal y Ecología, Universidad de Sevilla, 41012 Sevilla, Spain; 2 Departamento de Ciencias Agroforestales, Escuela Técnica Superior de Ingenierías Agrarias, Universidad de Valladolid (UVA), Palencia, Spain; 3 Instituto Universitario de Investigación en Gestión Forestal Sostenible, UVA, Palencia, Spain; 4 Área de Microbiología, Facultad de Medicina, Universidad de Castilla-La Mancha, Ciudad Real, Spain; 5 Grupo de Gestión de Recursos Cinegéticos y Fauna Silvestre, Instituto de Investigación en Recursos Cinegéticos (IREC, CSIC-UCLM-JCCM), Ciudad Real, Spain; 6 Departmento de Botánica, Ecología y Fisiología Vegetal, Universdad de Córdoba, 14071 Cordoba, Spain

**Keywords:** Ecological epidemiology, Population dynamics

## Abstract

Iberian hare populations have suffered severe declines during recent decades in Spain. Between 1970 and 1990s, a rapid increase in irrigation crop surface in NW Spain (Castilla-y-León region) was followed by a common vole massive range expansion and complete colonization of lowland irrigated agricultural landscapes from mountainous habitats. The subsequent large cyclic fluctuations in abundance of colonizing common voles have contributed to a periodic amplification of *Francisella* *tularensis*, the etiological agent that causes human tularemia outbreaks in the region. Tularemia is a fatal disease to lagomorphs, so we hypothesize that vole outbreaks would lead to disease spill over to Iberian hares, increasing prevalence of tularemia and declines among hare populations. Here we report on the possible effects that vole abundance fluctuations and concomitant tularemia outbreaks had on Iberian hare populations in NW Spain. We analysed hare hunting bag data for the region, which has been recurrently affected by vole outbreaks between 1996 and 2019. We also compiled data on *F.* *tularensis* prevalence in Iberian hares reported by the regional government between 2007 and 2016. Our results suggest that common vole outbreaks may limit the recovery of hare populations by amplifying and spreading tularemia in the environment. The recurrent rodent-driven outbreaks of tularemia in the region may result in a "disease pit" to Iberian hares: at low host densities, the rate of population growth in hares is lower than the rate at which disease-induced mortality increases with increased rodent host density, therefore, keeping hare populations on a low-density equilibrium. We highlight future research needs to clarify tularemia transmission pathways between voles and hares and confirm a disease pit process.

## Introduction

**Figure 1:**
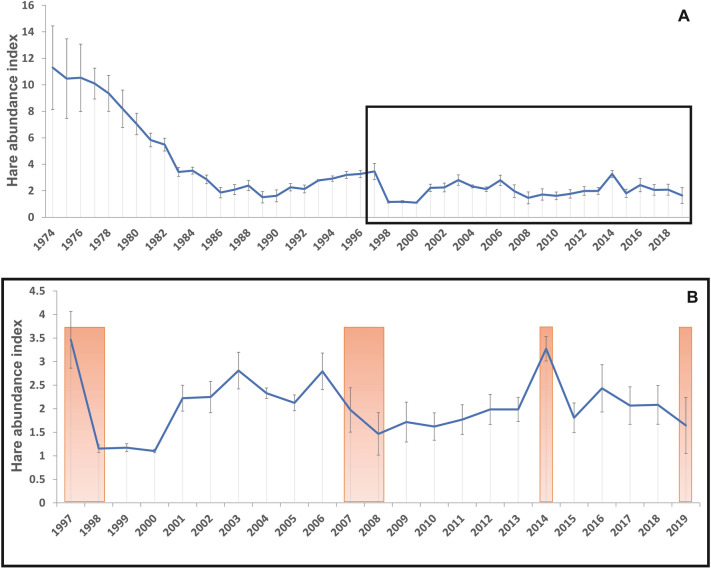
Iberian hare population trends. (**A**) Iberian hare abundance index (AI: number of hares hunted/number of hunting licences ± standard error) per year from 1974 to 2019. (**B**) Iberian hare abundance index during the study period (1997 to 2019). Brown bars indicate years when vole outbreaks were detected. Note that 1997 is the first year with officially declared human case of tularemia in the region.

Tularemia is an endemic zoonosis of the northern hemisphere caused by *Francisella* *tularensis*, with *F.* *tularensis* subsp. holarctica (Type B) being the only subspecies described in Europe^1^. In northwest (NW) Spain (i.e., Castilla-y-León region), tularemia is an emerging disease since 1997. Two large (regional scale) human tularemia outbreaks have been declared in Spain during 1997–1998 and 2007–2008, with over 1000 confirmed human cases^2^. Ever since 1997, all human tularemia epidemics have been linked to synchronous common vole (*Microtus* *arvalis*) population outbreaks in the area^3,4^. Rodents and lagomorphs, mainly hares, are the main putative mammalian reservoir hosts for *F.* *tularensis*^5,6^. In Spain and Portugal, the Iberian hare (*Lepus* *granatensis*) is an endemic species and is an important prey for many endangered predators such as the Spanish imperial eagle (*Aquila* *adalberti*) or the Iberian lynx (*Lynx* *pardinus*)^7^. Moreover, it is one of the most important small game species in Spain (i.e., ca.1 million hares are harvested annually during the hunting season^8^). However, Iberian hare populations have undergone sustained and severe declines, likely due to a combination of different factors such diseases, overhunting, habitat loss and agricultural intensification^9–11^. In Castilla-y-León, historic hunting records showed that Iberian hare populations dramatically declined between 1974 and 1986 (Fig. 1A, source Junta de Castilla-y-León, CAZDATA Project, https://medioambiente.jcyl.es/web/es/caza-pesca/cazdata-banco-datos-actividad.html [Cited 2022 Jun 10]). These population declines of hares coincided in time with profound land-use changes in the region, including a progressive increase of irrigation infrastructures. The increase in irrigated crop surface and the production of perennial fodder crops, especially alfalfa, favoured the rapid colonization by common vole populations^4,12,13^. From 1986 onwards, hare populations started a slow recovery, until 1997 when populations declined again. This crash concurred with a large regional population outbreak of voles and with the detection of the first cases of tularemia among humans^4^. From 1997 the Iberian hare populations have fluctuated but never recovered to earlier abundances (Fig. 1B). Here, we assess the possible effect that vole abundance fluctuations, and concomitant tularemia outbreaks, had on Iberian hare populations. For this purpose, we used hunting bag data as an index of hare abundance to calculate yearly hare population growth in Castilla-y-León region. We predicted increases in the prevalence of *F.* *tularensis* among Iberian hares during vole outbreak years, when tularemia prevalence increases in voles and contributes to disease spill-over and a massive environmental contamination^4,14^. Since tularemia increases mortality in hares^15^, we further expected hare population declines to occur during vole outbreaks years. We discuss our findings as potential evidence for a “disease pit” process that may be preventing hare population recovery. We also discuss alternative explanations for the observed patterns, in particular the potential indirect effects that vole predators and vole control measures (i.e., poisoning campaigns) might have had on hare populations.

## Methods

### Study site

Our study site is in an intensive agricultural landscape in NW Spain known as “Tierra de Campos”, which occupies part of three out of nine provinces of Castilla-y-León region (Palencia, Valladolid, and Zamora). This area is considered the main “hot-spot” of tularemia in Spain and Southern Europe^16^ and is characterized by higher-than-average vole abundances during outbreaks^17^.

### Iberian hare abundance index

Yearly occurrence of vole outbreaks in NW Spain between 1996 and 2020 (i.e., 1997, 1998, 2007, 2008, 2014, 2019) were identified based on reports in the news (historical reconstruction^18^) and more recently (from 2009 onward) using common vole abundance indices obtained from live-trapping monitoring (i.e.^4,19^).

To study the Iberian hare population trends we used regional hunting statistics available from the regional government (Junta de Castilla-y-León, CAZDATA Project, https://medioambiente.jcyl.es/web/es/caza-pesca/cazdata-banco-datos-actividad.html [Cited 2022 Sep 23]), which included hunting records as well as the number of hunting licences from 1974 to 2020. We used the number of hunted hares divided by the number of hunting licences each year as an abundance index for hares in “Tierra de Campos” (compiling data from the provinces of Palencia, Zamora and Valladolid). CAZDATA Project is an initiative proposed by the Hunting Federation of Castilla y León, which has the support of the regional government and, more importantly, the commitment of almost 60% of the hunting societies in the community to implement a system for monitoring hunting activity. Since this information is gathered by hunters for the benefit of the hunting activity, we are confidence on its reliability to carry out the present study.

### *Francisella tularensis* prevalence in Iberian hares

We compiled data on *F.* *tularensis* prevalence in Iberian hares from 2007 to 2016 using previously published information from a passive surveillance program carried out by the “Regional Network of Epidemiological Surveillance” (Red de Vigilancia Epidemiológica de la Dirección General de Salud Pública) of Castilla-y-León region^20^. This provided us with information on hare tularemia prevalence (amount of positives/number of screened individual) each year within the three provinces from “Tierra de Campos”.

### Statistical analyses

To study Iberian hare population trends, we calculated an index of yearly hare population instantaneous growth rate (PGR) using the hunting bag data (hare abundance index) from 1996 to 2020. Hare PGR was calculated as follows:

$$PGR= ln\left(\frac{{AI}_{t}}{{AI}_{t+1}}\right)$$

where *ln* stands for natural logarithm, *AI*_*t*_ is Iberian hare abundance index on year *t*. and *AI*_*t*+*1*_ is the Iberian hare abundance index on year *t* + *1*. PGRs were estimated yearly from 1996 to 2019. This dependent variable was fitted to a Generalized Linear Mixed Model using the *glmmTMB* function (GLMMTMB, package glmmTMB^21^) and a gaussian family distribution and identity link function. The categorical variable vole outbreak year (i.e., with two levels: years with (1) or without vole outbreak (0), hereafter “Vole”) and “Province” (i.e., with three levels: Palencia, Valladolid and Zamora), and their interaction were used as explanatory variables. “Year” of sampling was included as a random factor (i.e., 1996–2019). Significance of the fixed effects in the models was calculated with Type II tests using the function *Anova* in the car package^22^. We previously checked the model for overdispersion and distribution fitting using function *simulateResiduals* (package DHARMa^23^, simulations = 999). The variable PGR expresses the change between year *t* and *t* + *1*. We included *AI* at *t* as a covariate in the model, in order to take into account density-dependence in hare PGR (the extent to which the abundance changes in between year *t* and *t* + *1* depends on the abundance during year *t)*. For this to make biological sense, we rescaled the covariable *AI* so that it has mean equal to zero. Thus, the effect of the other predictor variables in the model (i.e., “Vole” and “Province”) was interpreted as the effect that these variables have on PGR when the abundance value is at 0. Thus, the effect of “Vole” and “Province” on PGR will be obtained by the mean value of abundance.

We assessed the effect of vole outbreak years on the Iberian hare’s population PGR by running a multiple Pearson correlation (function *ggscatter*) between PGR and *AI*, considering both, PGR for all the years of the study period (i.e., 1996–2019) and only those years where vole outbreaks were detected (i.e., 1997, 1998, 2007, 2008, 2014, 2019).

Finally, we tested for difference in the prevalence of *F.* *tularensis* on Iberian hare’s during years with or without vole outbreaks using a GLMMTMB^21^ with a binomial family distribution and a logit link function, where prevalence of *F.* *tularensis* in hares was the dependent variable, and “Vole” outbreak years and “Province” (i.e. Palencia, Valladolid and Zamora) were the responses variables. In this case, the variable “Vole” outbreak years included three levels (i.e. 0 = no vole outbreak, 1 = vole outbreak year, 2 = one year after vole outbreak), to assess if *F.* *tularensis* prevalence in hare also persist one year after a vole outbreak. “Year” of sampling was included as a random factor (i.e., 2007–2016). Due to the limited sample size, we did not include the interaction between “Vole” and “Province” to not overfit the model. We also previously checked the model for overdispersion and distribution fitting using function *simulateResiduals* (package DHARMa^23^, simulations = 999). All analysis were carried out using the R statistical computing environment^24^.

## Results

**Figure 2:**
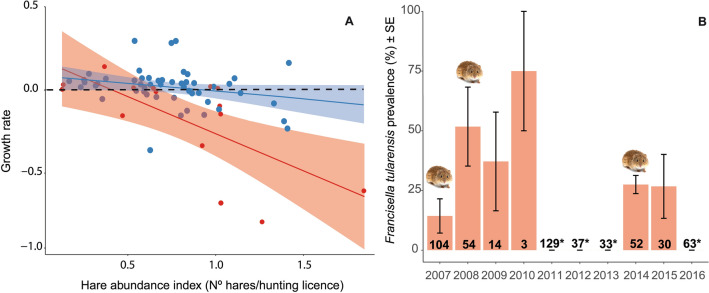
(**A**) Relationships between the Iberian hare population instantaneous growth rate (PGR) and the hare abundance index (AI, number of hunted hares/hunting licences per year) during vole outbreak years (in red) and during all years (in blue). (**B**) Prevalence (% ± standard error) of *Francicela* *tularensis* in Iberian hares collected through passive surveillance from 2007 to 2016. Numbers in bold shows sample size (number of hares screened for tularemia). For some years marked with an asterisk (*), numbers refer to both Iberian hares and wild rabbit (lumped together in the data provided by Rodríguez-Ferri (2017)^20^. Note that *F.* *tularensis* prevalence during those years was zero, so uncertainty regarding hare sample size was unlikely to affect conclusions. In addition, the mean prevalence of *F.* *tularensis* in 2010 was obtained from a total of three samples; one positive hare in the province of Zamora and another positive hare from two hares collected in the province of Valladolid. Although the mean is represented in the graph, this low sample size makes us believe that it is not representative of the real prevalence.

During years of common vole outbreaks, our yearly index of Iberian hare abundance (hunting bags corrected for the number of hunting licenses) was on average 8% lower than during other years. Iberian hare instantaneous population growth rate (PGR) was also significantly lower than during years without vole outbreaks (*β* = −0.184, SE = 0.044, Z = 4.149, *P* < 0.0001), a difference that was consistently found in the three provinces studied (Table 1, Fig. 1S). Pearson multiple correlation revealed a negative association between Iberian hare PGR and Iberian hare abundance index (Fig. 2A), typical of a density-dependence relationship (populations tend to grow from lower density, and to decline from higher density). However, the slope of this correlation differed between years with or without vole outbreak. The negative density-dependent relationship was steeper during years with vole population outbreaks (R = −0.69, *P* = 0.0044) than during other years (R = −0.24, *P* = 0.083) (Fig. 2A).

**Table 1:** Results of generalized linear mixed model using template model builder (GLMMTMB) to study the effect of common vole (*Microtus* *arvalis*) outbreaks years on the growth rate of Iberian hare (*Lepus* *granatensis*) populations in Tierra de Campos (NW Spain) and accounting for the different provinces.

Fixed effects	Estimate	S.E.	Type II Wald *χ*^*2*^	*df*	*P*
Intercept	0.113	0.038	–		–
Vole	–	–	17.71	1	** < 0.0001**
Vole (outbreak)	−0.184	0.044	–		–
Province	–	–	17.02	2	**0.0002**
Province (Valladolid)	0.177	0.047	–	–	–
Province (Zamora)	0.179	0.047	–	–	–
Vole: Province	–	–	0.64	1	0.72
AI	–	–	30.23	1	** < 0.0001**

Random effects	Variance	Std. dev			
Year	0.0015	0.038			
Residuals	0.017				

Iberian hare abundance index (AI) was included as a covariate in the model.

Significant values are in bold.

Tularemia prevalence in hares collected during passive surveillance between 2007 and 2016 varied among years and was significantly higher (ca. threefold) during vole outbreak years than during other years (*β* = 4.531, SE = 0.812, Z = 5.576, *P* < 0.0001, Fig. 2B, Table 2). Moreover, *F.* *tularensis* prevalence in hares was significantly higher (ca. 3-times higher) one year after the vole population outbreaks (*β* = 4.798, SE = 0.853, Z = 5.624, *P* < 0.0001, Fig. 2B, Table 2), when vole populations had crashed.

**Table 2:** Results of generalized linear mixed model using template model builder (GLMMTMB) to study the effect of common vole (*Microtus* *arvalis*) outbreaks years (i.e. Vole 1) and 1 year after (Vole 2) on the prevalence of *Francisella* *tularensis* on of Iberian hare (*Lepus* *granatensis*) populations in Tierra de Campos (NW Spain) and accounting for the different provinces.

Fixed effects	Estimate	S.E.	*Z-*value	*P*
Intercept	−5.534	0.836	−6.619	** < 0.0001**
Vole 1	4.531	0.812	5.576	** < 0.0001**
Vole 2	4.798	0.853	5.624	** < 0.0001**
Province (Valladolid)	0.152	0.328	0.464	0.643
Province (Zamora)	0.807	0.446	1.807	0.071

Random effects	Variance	Std. dev		
Year	0.089	0.299		

Note that intercept included Palencia province.

Significant values are in bold.

## Discussion

In this study we carried out a detailed analysis of the Iberian hare population trend starting just before the first outbreak of tularemia recorded in Spain during 1997. Our results revealed a reduced PGR of the Iberian hare population during years of common vole outbreaks in comparison with other years. Moreover, hare PGR was lower than expected from current density during years with vole outbreaks, possibly because of increased mortality. We also detected that *F.* *tularensis* prevalence in hares was significantly higher during both, years of vole outbreak and one year after a vole outbreak was detected. This suggested that *F.* *tularensis* persisted in the hare population and/or the environment for at least one year after vole outbreaks. These results provide correlative evidence for an indirect impact of vole population outbreaks on the Iberian hare population of Tierra de Campos (NW Spain). In fact, *F.* *tularensis* disease transmission and spill over contamination of the environment has been shown to be greatly enhanced by high vole densities^3,16^. This is because in the study area *F.* *tularensis* prevalence has a direct density-dependent relation with vole abundance^25^: the higher the vole abundance, the higher the prevalence of *F.* *tularensis,* which reached up to 37% and 33% prevalence during the 2007^25^ and 2014^26^ vole population peaks, respectively. Previous works showed that spill-over contamination to humans occurred during vole outbreak years^3,4,16^, and our results suggests that spill-over contamination likely also affected Iberian hares in the region. *Francisella* *tularensis* infection causes increased mortality in hares, although individuals may respond differently to the infection. Some hares may die of overwhelming bacteraemia, others may survive with a protracted course of infection^27^, which would contribute to the maintenance of *F.* *tularensis* prevalence. This is consistent with the view that hares act as key reservoirs in this system: while voles cause pulses of tularemia contamination during outbreaks, hares may play a key role in maintaining the bacterium, at least for one more year after a vole peak, since hares eventuality decline after the outbreak. Tularemia was not detected in hares during inter-epizootics beyond the second year of an outbreak, so other environmental reservoirs must be involved in the tularemia cycle, contributing to the persistence of *F.* *tularensis* in the environment. A prolonged survival of the pathogen may occur outside hosts, for example in water ^28^, or in invertebrate vectors (e.g., ticks)^29^ that might aid infection at a later point, when vole numbers increase again.

Regardless of where the pathogen persists during the interepizootic periods, our results suggest that *F.* *tularensis* spill-over, facilitated by the vole population outbreaks, contributed to a reduced hare population growth rate. Such process may be “trapping” the hare’s population into a locally stable state of low density in the study area (i.e., a “diseases pit” scenario), keeping hare numbers underneath a threshold abundance around 4 hares/hunter (Fig. 1B). This situation arises at low prey densities when the rate of population growth is lower than the rate at which diseases-induced mortality increases with increased prey density. A similar “disease pit” situation has been suggested for European wild rabbits (*Oryctolagus* *cuniculus*) in Doñana National Park DNP (southern Spain), where after a sudden population crash due to the impact of rabbit haemorrhagic disease (RHD) in the 1990s, rabbit numbers should have kept stable in DNP but at low densities due to predator regulation (i.e., “predator pit”^30,31^). However, RHD is still killing susceptible animals recruited each year after reproduction in DNP, and consequently the population is progressively declining^32^. There are alternative explanations to the observed hare declines during vole outbreak years. First, hares might have declined because of vole control actions, for example, as collateral damage of rodenticide use (e.g., bromadiolone or chlorophacinone^25,33^). While this probably contributed to a higher hare mortality during the 2007–08 outbreaks, when the use of bromadiolone was allowed at large-scales, it is unlikely to explain the hare population decline during the subsequence vole outbreaks, since anticoagulant rodenticide use was prohibited afterwards. A second alternative explanation of hare declines after vole outbreaks could be the impact of predators attracted by sudden prey increases in the landscape (i.e., common voles). However, increases in generalist vole predators usually occur with a time delay (it takes time for vole predators to breed). This last alternative explanation seems unlikely given that Iberian hare population declines occurred during vole outbreak years (without lag), when there were still plenty of voles for both specialists and generalist predators to feed on. Because diseases are difficult to manage or control in the wild, any effective future effort to increase Iberian hare populations should be based on reliable information that includes simultaneous data on population dynamics and field epidemiology. Future work is needed to confirm the “disease pit” hypothesis proposed here. In the absence of a tularaemia vaccine for wildlife, we suggest that the most informative approach would be a detailed longitudinal study of tularemia infection among Iberian hares. Tagging hares will allow to determine demographic parameters, such as survival and reproduction, and relate these to tularemia infections. It would also be useful to compare the demographic parameters of hares in areas with and without *F.* *tularensis* to verify if the recruitment is higher than the mortality rate where tularemia is absent, and thereby obtain empirical evidence supporting a “disease pit” scenario.

## Supplementary Information

Supplementary Figure S1.

## Data Availability

The data that support the findings of this study are available from the corresponding author upon reasonable request.
