# Supplementary material for: Vole outbreaks may induce a tularemia disease pit that prevents Iberian hare population recovery in NW Spain
Source: Sci Rep. 2023 Mar 8;13:3898. doi: 10.1038/s41598-023-30651-7 (PMC9995447; doi:10.1038/s41598-023-30651-7)
Supplement: Supplementary file 1 — Supplementary Figure S1. [file 41598_2023_30651_MOESM1_ESM.docx]

SUPPLEMENTARY MATERIAL


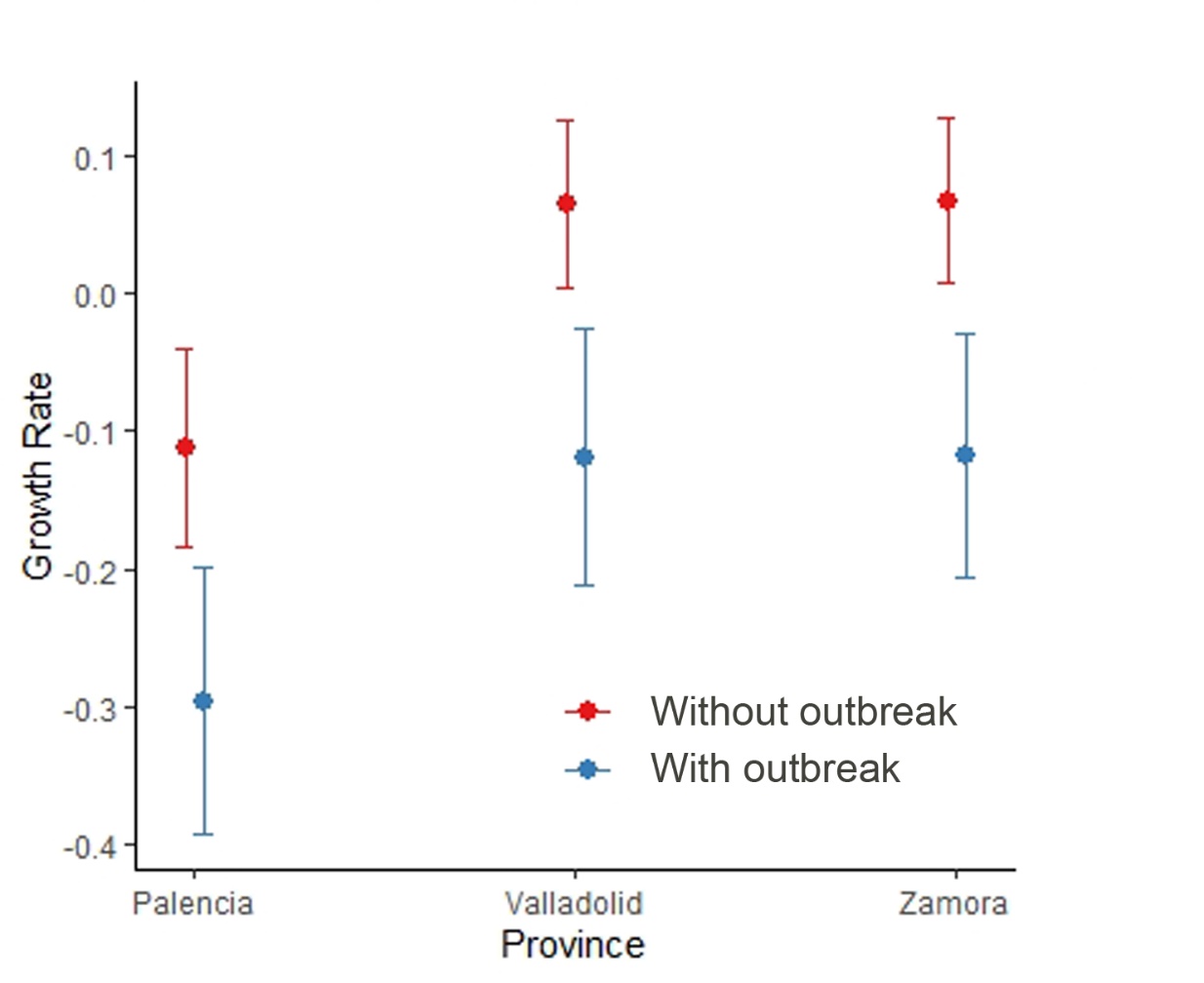


**Figure 1S.** Average growth rate (95% Confidence Intervals) of the Iberian hare (*Lepus granatensis*) populations on each province of Tierra de Campos (i.e. Palencia, Valladolid and Zamora) during years with common vole (*Microtus arvalis*) population outbreaks (With outbreaks) and without them (Without outbreaks) between 1996 and 2019.
